# Supplementary figures and images for: Genomic ascertainment of CHEK2-related cancer predisposition
Source: medRxiv. 2024 Aug 8:2024.08.07.24311613. Preprint. [Version 1] doi: 10.1101/2024.08.07.24311613 (PMC11451703; doi:10.1101/2024.08.07.24311613)

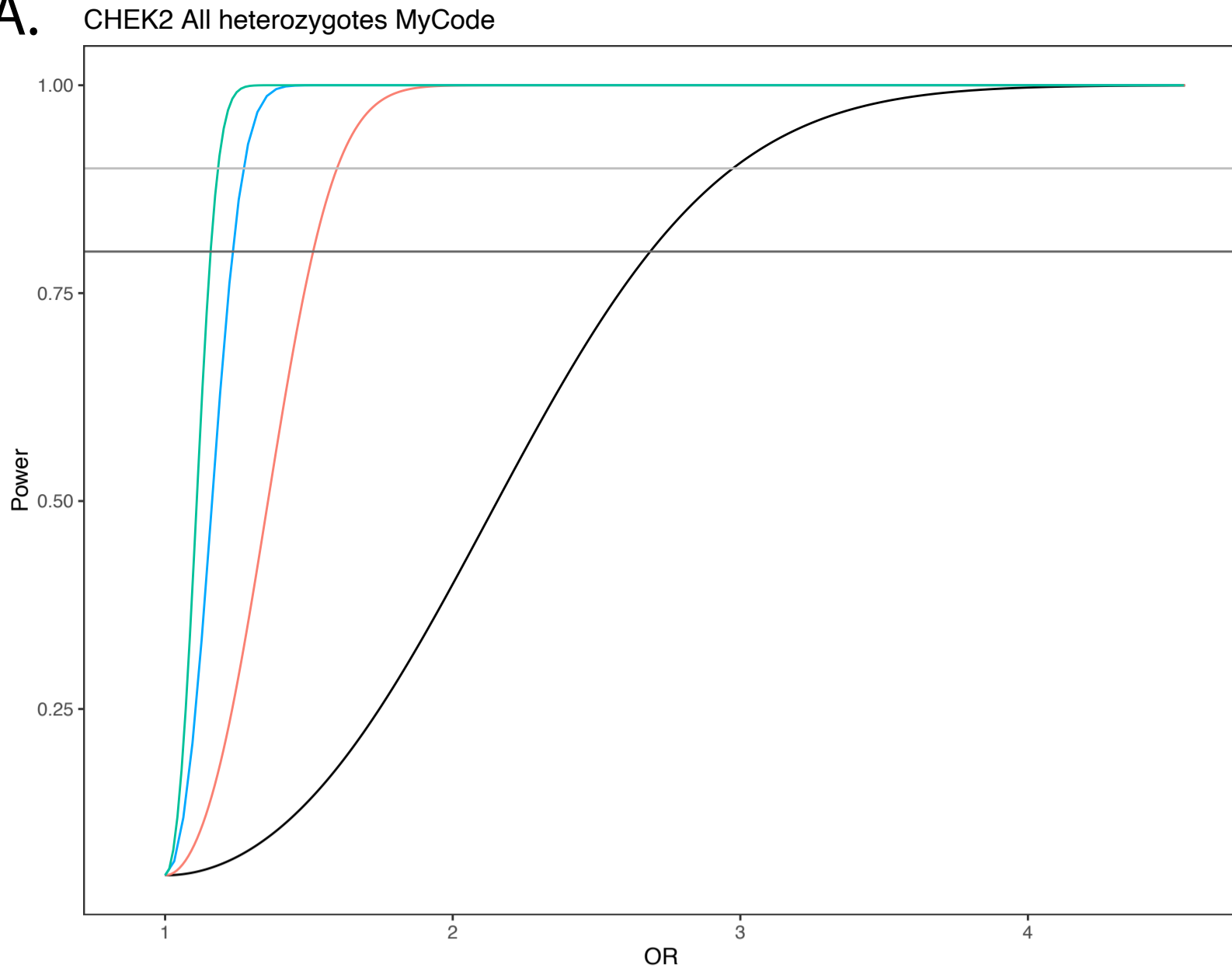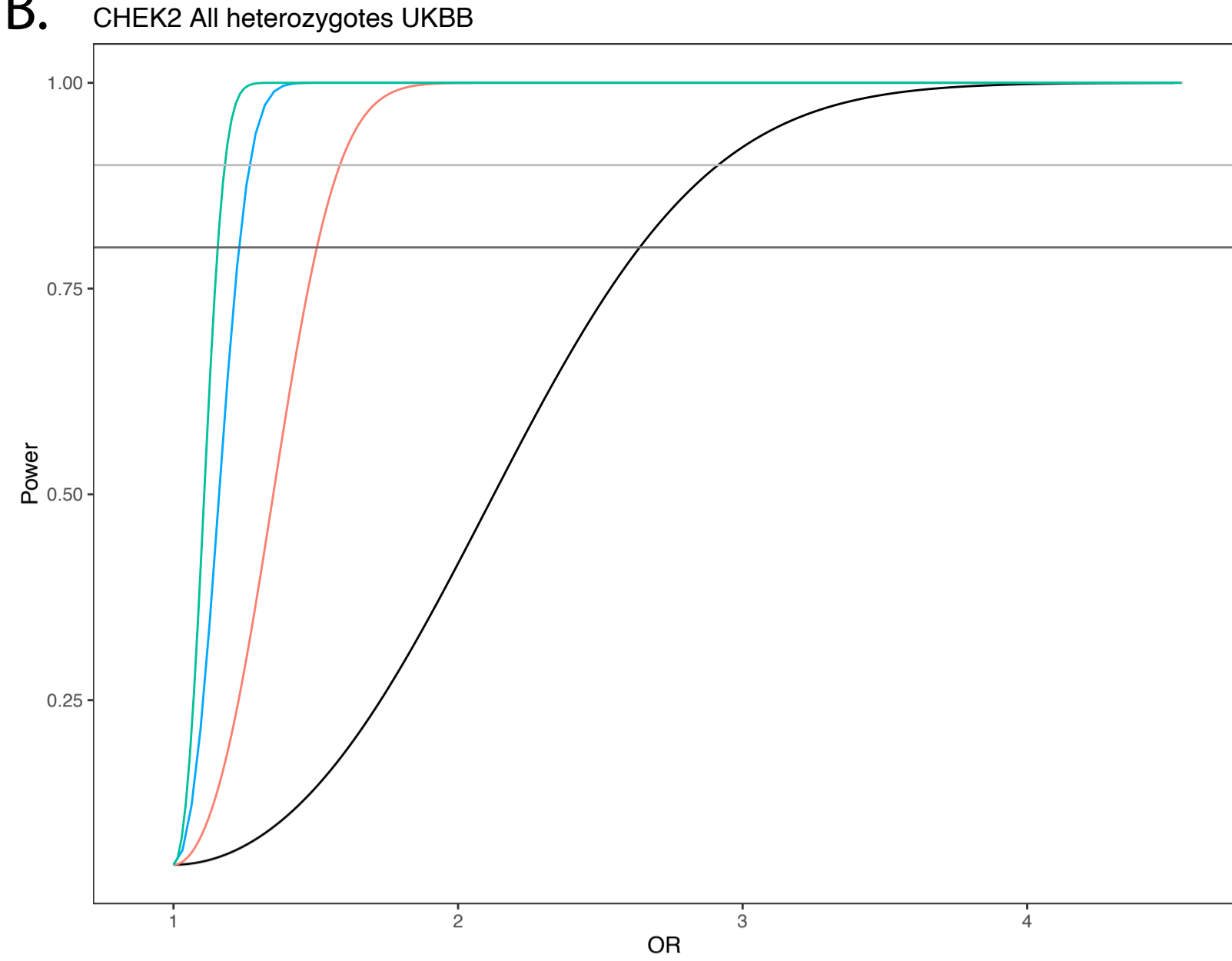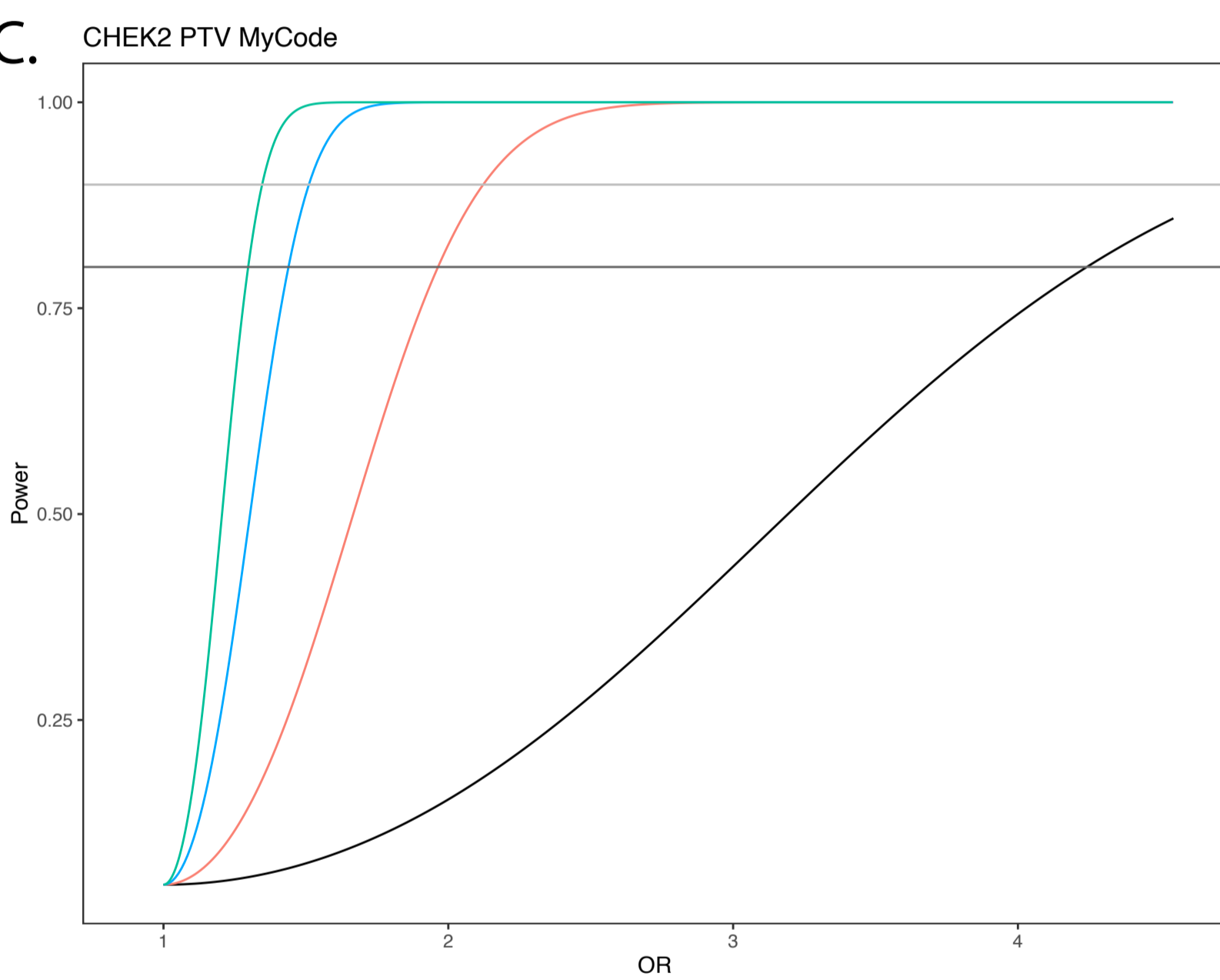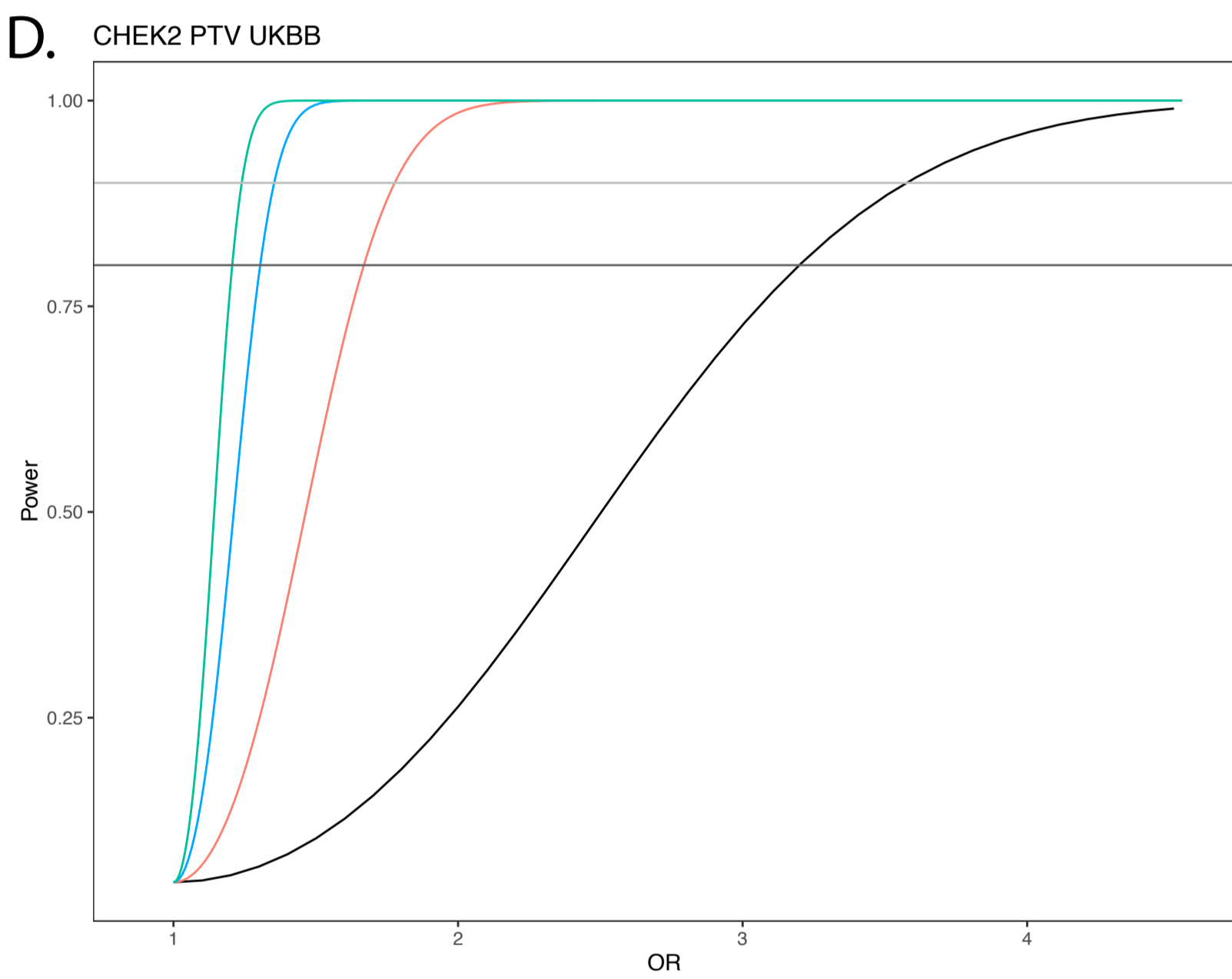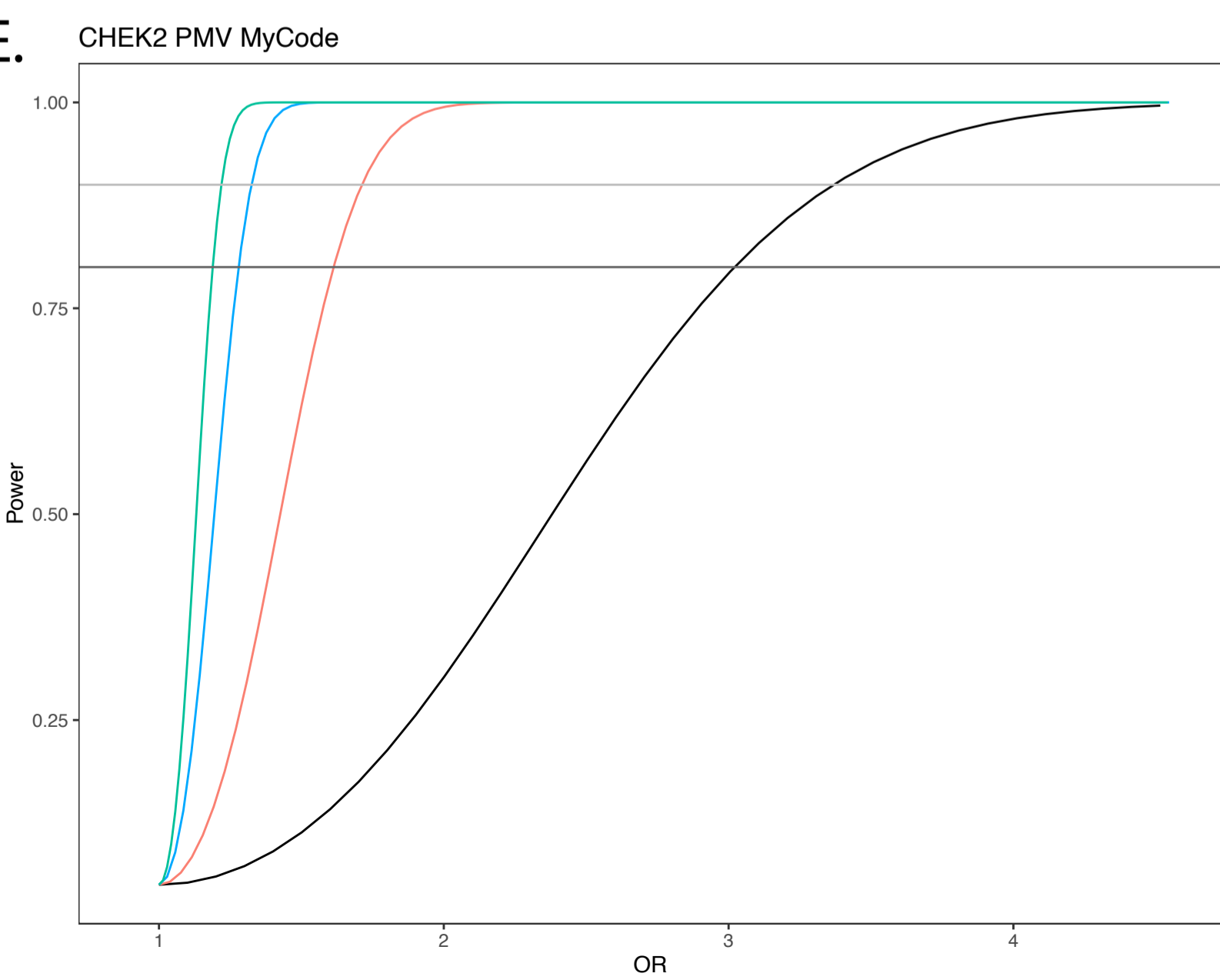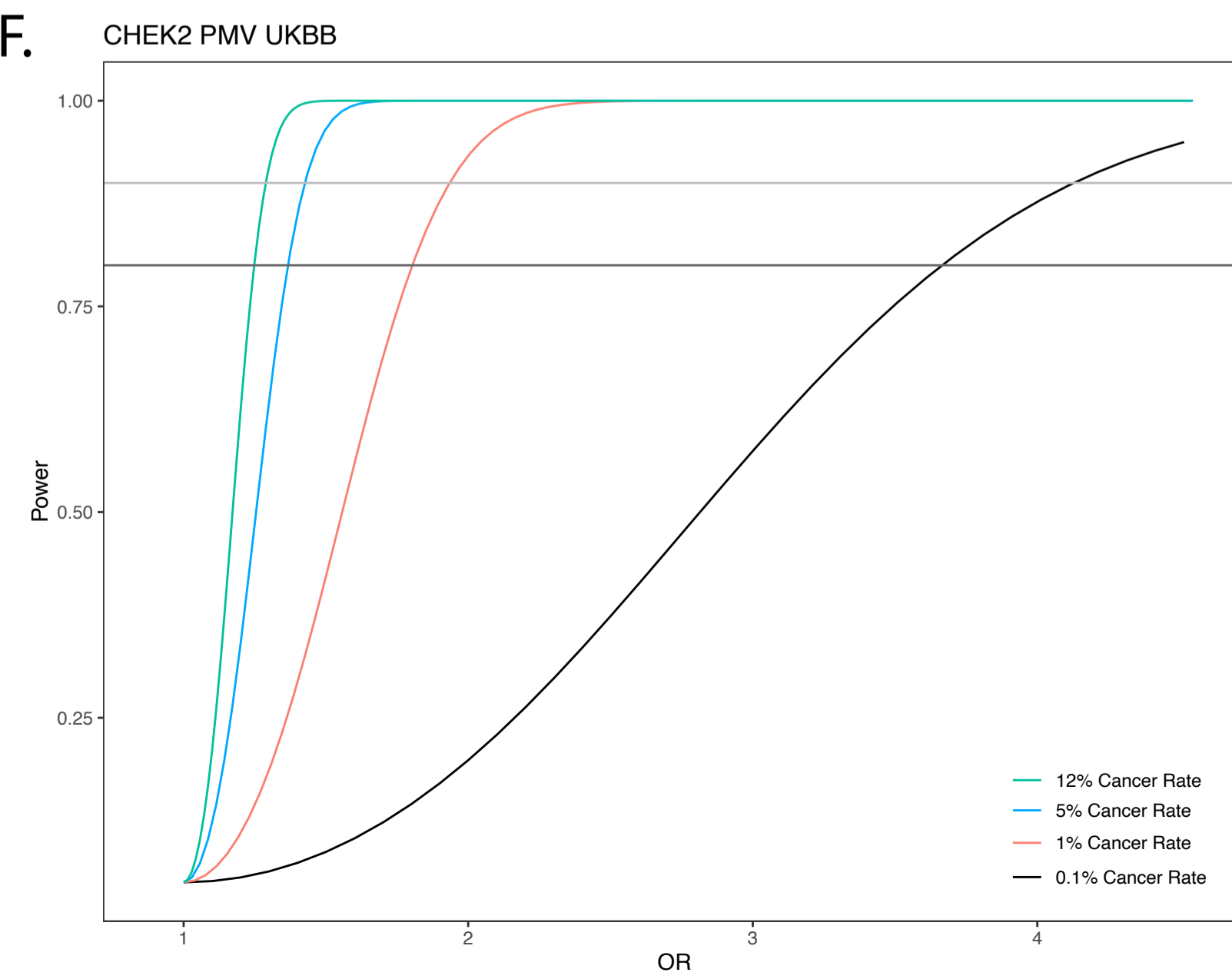

Supplement: Supplement 1 — Supplemental Figure 1. Power as a function of risk (odds ratio) in MyCode (Panel A, C, E) and UK Biobank (Panels B, D, F) for a range of cancer rates. Prevalence data from cohort-specific ALL (Panels A, B) pathogenic truncating variants (PTV) (Panels C, D) and pathogenic missense variants (PMV) (Panels E, F) CHEK2 heterozygotes (Table 1). Dark gray line represents 80% power, and light gray line represents 90% power. [file media-1.pdf]

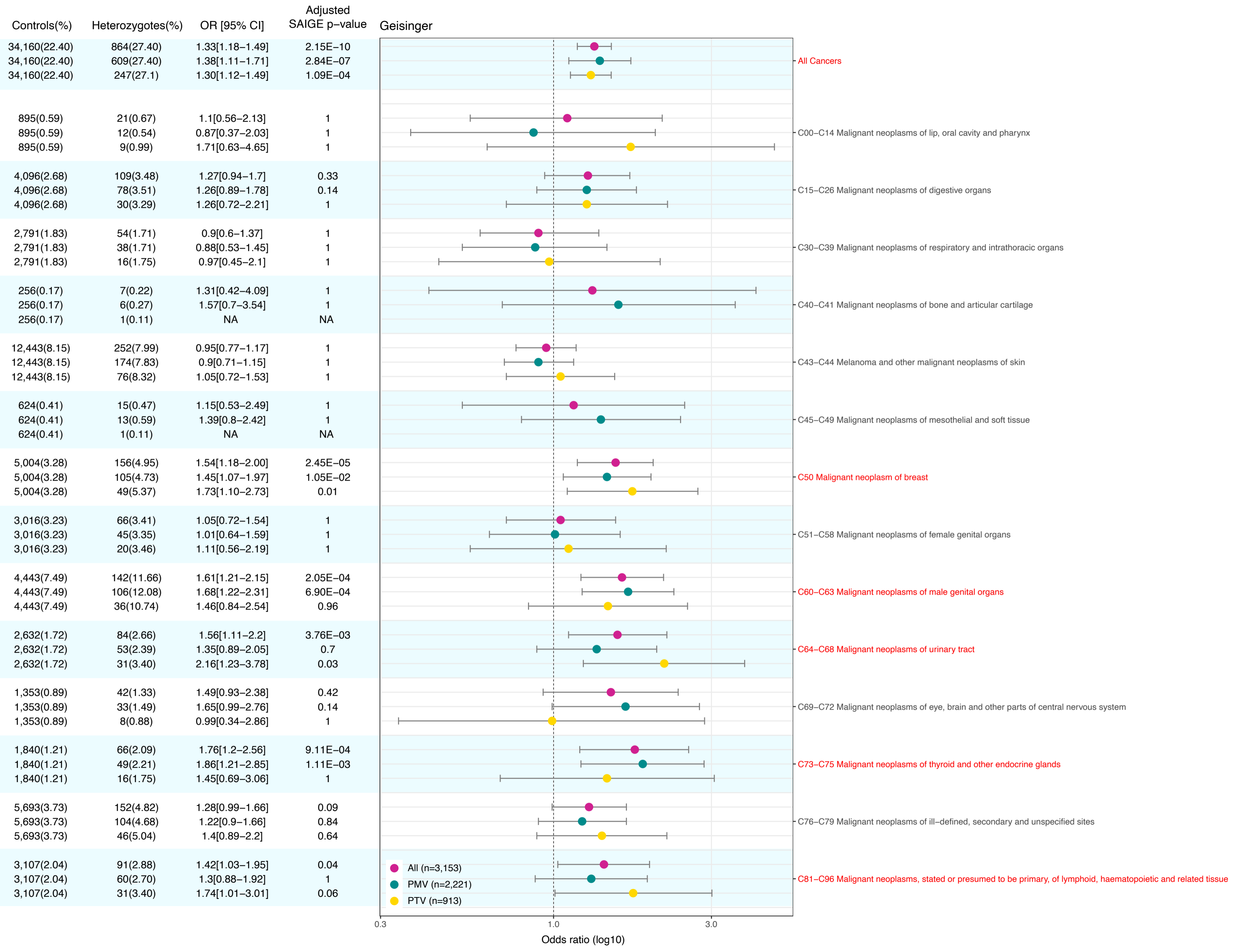

Supplement: Supplement 2 — Supplemental Figure 2. Odds ratio for All, PTV and PMV CHEK2 heterozygotes for organ system groupings of cancer ICD codes in MyCode. Red font represents significant cancers. CI: 95% confidence interval; OR: odds ratio; PMV: pathogenic missense variant; PTV: pathogenic truncating variant [file media-2.pdf]

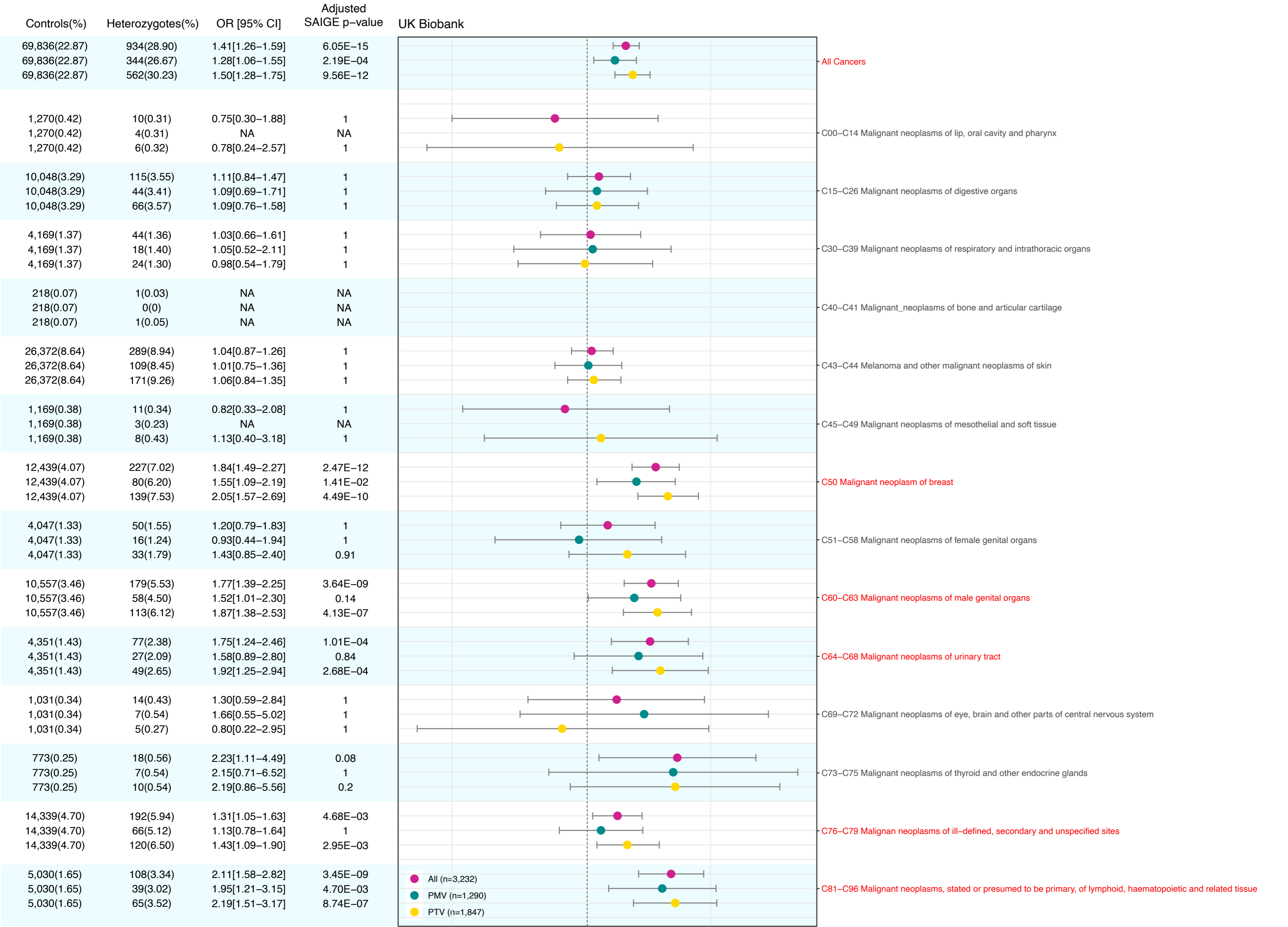

Supplement: Supplement 3 — Supplemental Figure 3. Odds ratio for All, PTV and PMV CHEK2 heterozygotes for organ system groupings of cancer ICD codes in UK Biobank. Red font represents significant cancers. CI: 95% confidence interval; OR: odds ratio; PMV: pathogenic missense variant; PTV: pathogenic truncating variant [file media-3.pdf]

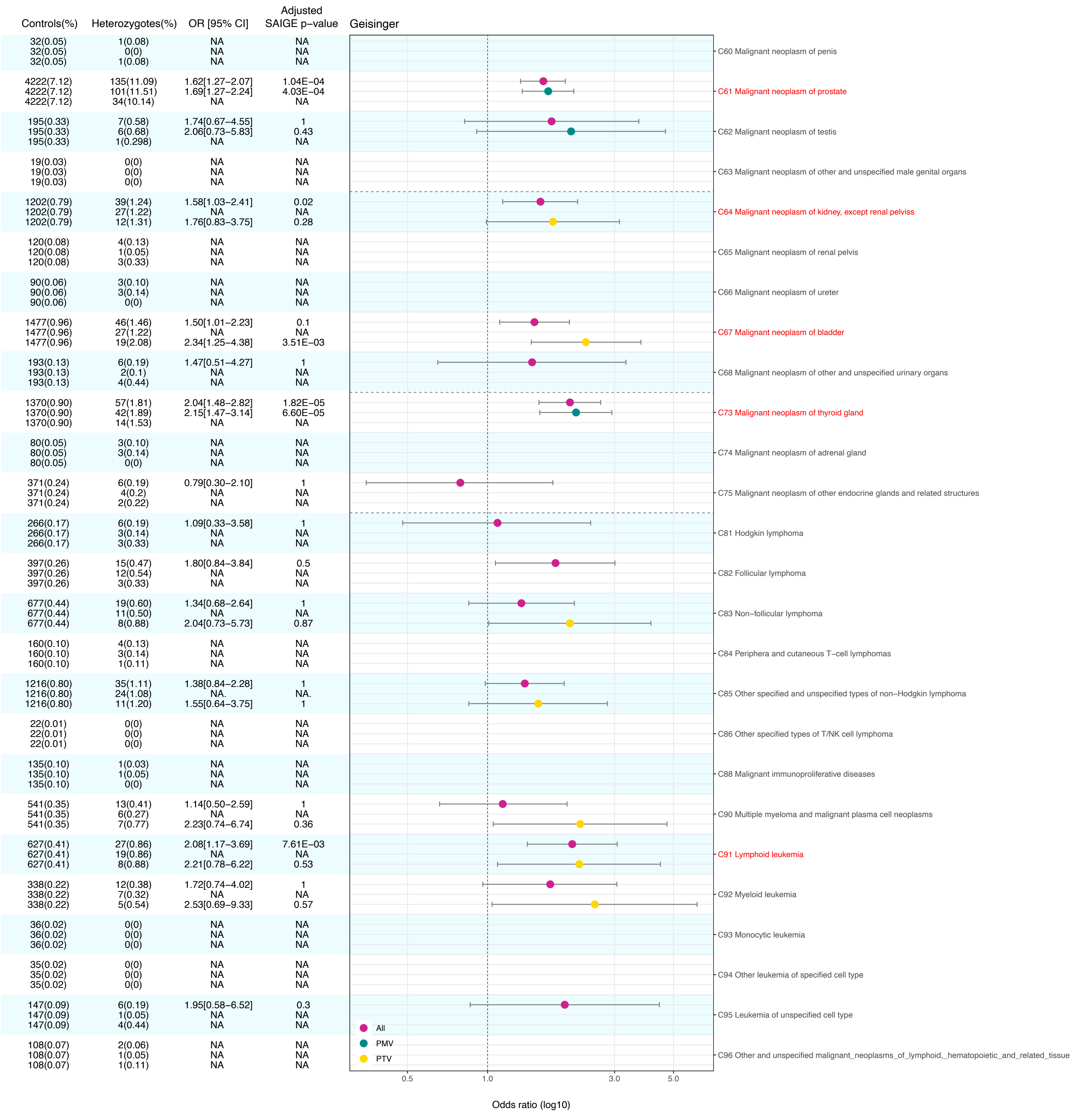

Supplement: Supplement 4 — Supplemental Figure 4. Odds ratio for All, PTV and PMV CHEK2 heterozygotes for all specific cancers in the organ system groupings of cancer ICD codes in MyCode. Red font represents significant cancers. CI: 95% confidence interval; OR: odds ratio; PMV: pathogenic missense variant; PTV: pathogenic truncating variant [file media-4.pdf]
