# Supplementary material for: Genomic ascertainment of CHEK2-related cancer predisposition
Source: medRxiv. 2024 Aug 8:2024.08.07.24311613. Preprint. [Version 1] doi: 10.1101/2024.08.07.24311613 (PMC11451703; doi:10.1101/2024.08.07.24311613)

Controls(%)	Heterozygotes(%)	OR [95% CI]	Adjusted SAIGE p−value	
91(0.03)	0(0)	NA	NA	C60 Malignant neoplasm of penis
91(0.03)	0(0)	NA	NA	
91(0.03)	0(0)	NA	NA	
9,978(3.27)	170(5.27)	1.78[1.48–2.16]	6.68E−10	C61 Malignant neoplasm of prostate
9,978(3.27)	55(4.26)	NA	NA	
9,978(3.27)	107(5.79)	1.87[1.47–2.38]	1.27E−07	
517(0.17)	10(0.31)	1.82[0.89–3.72]	0.12	C62 Malignant neoplasm of testis
517(0.17)	3(0.23)	NA	NA	
517(0.17)	7(0.38)	2.16[0.92–5.09]	0.15	
22(0.01)	0(0)	NA	NA	C63 Malignant neoplasm of other and unspecified male genital organs
22(0.01)	0(0)	NA	NA	
22(0.01)	0(0)	NA	NA	
1,635(0.54)	31(0.96)	1.84[1.22–2.77]	2.49E−03	C64 Malignant neoplasm of kidney, except renal pelvis
1,635(0.54)	11(0.85)	NA	NA	
1,635(0.54)	20(1.08)	2.05[1.23–3.40]	4.28E−03	
142(0.05)	2(0.06)	NA	NA	C65 Malignant neoplasm of renal pelvis
142(0.05)	2(0.16)	NA	NA	
142(0.05)	0(0)	NA	NA	
163(0.05)	1(0.03)	NA	NA	C66 Malignant neoplasm of ureter
163(0.05)	1(0.08)	NA	NA	
163(0.05)	0(0)	NA	NA	
2,707(0.89)	45(1.40)	1.64[1.17–2.31]	2.66E−03	C67 Malignant neoplasm of bladder
2,707(0.89)	15(1.16)	NA.	NA.	
2,707(0.89)	30(1.62)	1.88[1.24–2.85]	2.32E−03	
86(0.06)	3(0.09)	NA	NA	C68 Malignant neoplasms of other and unspecified unrinary organs
86(0.06)	1(0.08)	NA	NA	
86(0.06)	2(0.11)	NA	NA	
175(0.06)	3(0.09)	NA	NA	C76 Malignant neoplasm of other and ill−defined sites
175(0.06)	1(0.08)	NA	NA	
175(0.06)	2(0.11)	NA	NA	
7,493(2.45)	109(3.38)	1.41[1.11–1.78]	9.77E−04	C77 Secondary and unspecified malignant neoplasm of lymph nodes
7,493(2.45)	40(3.10)	NA	NA	
7,493(2.45)	66(3.57)	1.50[1.11–2.03]	4.23E−03	
7,557(2.48)	94(2.92)	1.21[0.94–1.55]	0.21	C78 Secondary malignant neoplasm of respiratory and digestive organs
7,557(2.48)	32(2.48)	NA	NA	
7,557(2.48)	59(3.19)	1.32[0.96–1.81]	0.16	
6,072(2.00)	84(2.61)	1.34[1.03–1.76]	0.01	C79 Secondary malignant neoplasm of other sites
6,072(2.00)	27(2.09)	NA	NA	
6,072(1.99)	56(3.03)	1.56[1.13–2.17]	4.40E−03	
414(0.14)	8(0.25)	1.83[0.68–4.94]	0.02	C81 Hodgkin's disease
414(0.14)	0(0)	NA	NA	
414(0.14)	8(0.43)	3.19[1.22–8.36]	8.40E−03	
593(0.19)	10(0.31)	1.61[0.66–3.90]	1	C82 Follicular [nodular] non−Hodgkin's lymphoma
593(0.19)	5(0.39)	2.04[0.61–6.84]	1	
593(0.19)	5(0.27)	1.39[0.42–4.67]	1	
1,362(0.45)	26(0.81)	1.84[1.06–3.20]	0.03	C83 Diffuse non−Hodgkin's lymphoma
1,362(0.45)	7(0.54)	1.27[0.46–3.55]	1	
1,362(0.45)	18(0.97)	2.19[1.15–4.17]	0.01	
216(0.07)	6(0.19)	2.68[0.85–8.45]	0.24	C84 Peripheral and cutaneous T−cell lymphomas
216(0.07)	5(0.39)	5.76[1.70–19.50]	0.02	
216(0.07)	1(0.05)	NA	NA	
1,533(0.50)	29(0.90)	1.83[1.08–3.08]	0.02	C85 Other and unspecified types of non−Hodgkin's lymphoma
1,533(0.50)	8(0.62)	1.28[0.49–3.34]	0.94	
1,533(0.50)	20(1.08)	2.18[1.18–4.00]	0.02	
42(0.01)	4(0.12)	NA	NA	C86 Other specified types of T/NK−cell lymphoma
42(0.01)	3(0.23)	NA	NA	
42(0.01)	1(0.05)	NA	NA	
180(0.06)	4(0.12)	NA	NA	C88 Malignant immunoproliferative diseases
180(0.06)	1(0.08)	NA	NA	
180(0.06)	3(0.16)	NA	NA	
933(0.31)	18(0.56)	1.86[0.96–3.61]	0.09	C90 Multiple myeloma and malignant plasma cell neoplasms
933(0.31)	7(0.54)	1.87[0.67–5.20]	1	
933(0.31)	10(0.54)	1.77[0.75–4.19]	0.29	
921(0.30)	21(0.65)	2.21[1.19–4.08]	0.01	C91 Lymphoid leukaemia
921(0.30)	9(0.70)	2.44[0.99–6.05]	0.14	
921(0.30)	12(0.65)	2.16[0.99–4.74]	0.08	
650(0.21)	20(0.62)	2.97[1.58–5.58]	2.17E−04	C92 Myeloid leukaemia
650(0.21)	7(0.54)	2.66[0.95–7.41]	0.13	
650(0.21)	13(0.70)	3.32[1.56–7.08]	1.22E−04	
82(0.03)	2(0.06)	NA	NA	C93 Monocytic leukaemia
82(0.03)	2(0.16)	NA	NA	
82(0.03)	0(0)	NA	NA	
46(0.02)	3(0.09)	NA	NA	C94 Other leukaemias of specified cell type
46(0.02)	2(0.16)	NA	NA	
46(0.02)	1(0.05)	NA	NA	
103(0.03)	1(0.03)	NA	NA	C95 Leukaemia of unspecified cell type
103(0.03)	0(0)	NA	NA	
103(0.03)	1(0.05)	NA	NA	
50(0.02)	5(0.16)	9.47[2.58–34.80]	1.91E−03	C96 Other and unspecified malignant neoplasms of lymphoid, haematopoietic and related tissue
50(0.02)	3(0.23)	NA	NA	
50(0.02)	2(0.11)	NA	NA	

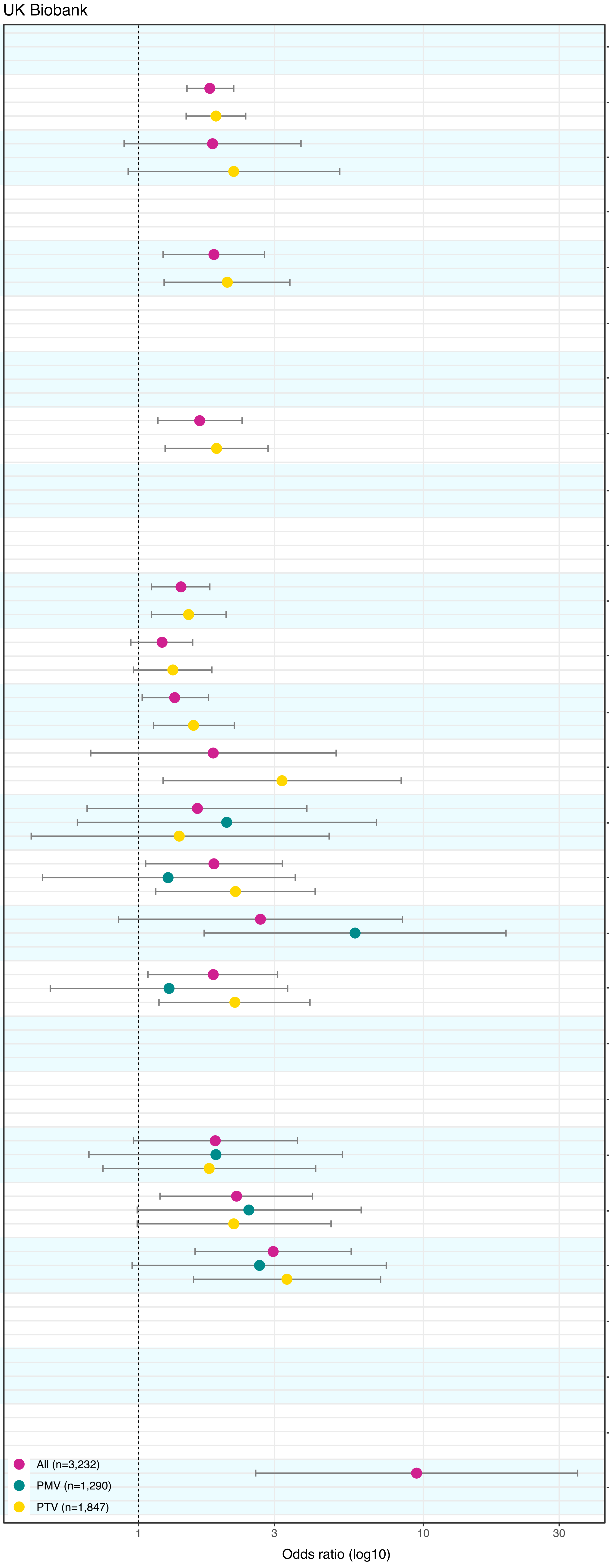

Supplement: Supplement 5 — Supplemental Figure 5. Odds ratio for All, PTV and PMV CHEK2 heterozygotes for all specific cancers in the organ system groupings of cancer ICD codes in UK Biobank. Red font represents significant cancers. CI: 95% confidence interval; OR: odds ratio; PMV: pathogenic missense variant; PTV: pathogenic truncating variant [file media-5.pdf]
